# Role of Sentinel Lymph Node Biopsy in the Management of Merkel Cell Carcinoma

**DOI:** 10.1155/2012/176173

**Published:** 2012-10-03

**Authors:** Eric P. Arruda, Kevin M. Higgins

**Affiliations:** ^1^Head and Neck Surgery, Department of Otolaryngology, University of Toronto, Toronto, ON, Canada M5G 2N2; ^2^Head and Neck Surgery, Department of Otolaryngology, Sunnybrook Health Sciences Centre, 2075 Bayview Avenue M1-102, Toronto, ON, Canada M4N 3M5; ^3^Division of Head and Neck Oncology, Odette Cancer Centre, Toronto, ON, Canada M4N 3M5

## Abstract

Merkel cell carcinoma (MCC) is a rare and typically aggressive form of skin cancer. It most commonly affects the elderly and has a predilection for the sun-exposed skin of the head and neck region. Other etiological factors include immune suppression, organ transplantation, and polyoma virus infection. MCC has a propensity to spread to regional lymphatics with a high locoregional recurrence rate. Since its discovery in 1972, treatment paradigms have shifted, with no consensus on optimal management strategies. Currently, standard of care includes surgical intervention to the primary and locoregional site with adjuvant radiotherapy for high-risk disease. In this paper, we discuss the history, pathology, and epidemiology of this rare disease with a focus on the evidentiary basis of treatment protocols. The use of sentinel lymph node biopsy as a management option will be the focus of this paper.

## 1. Introduction

Merkel cell carcinoma (MCC) is a rare and aggressive neoplasia first described in 1972 by Toker [1]. First described as trabecular carcinoma of the skin as a consequence of its column-like growth pattern, MCC currently has many synonyms including cutaneous neuroendocrine carcinoma, and small-cell primary cutaneous carcinoma [2]. The discovery of neurosecretory granules in three of the original tumours studied by electon microscopy raised the possibility of a neuroendocrine source, and the MC was proposed as the cellular origin [3]. It has been shown that MC and MCC have overlapping electron microscopic features and immunohistochemical profiles which support the MC as the cellular origin of this aggressive tumour. The term Merkel cell carcinoma was coined by DeWoolf-Peters in 1980 and today remains the most accepted terminology [4].

**Figure 1:**
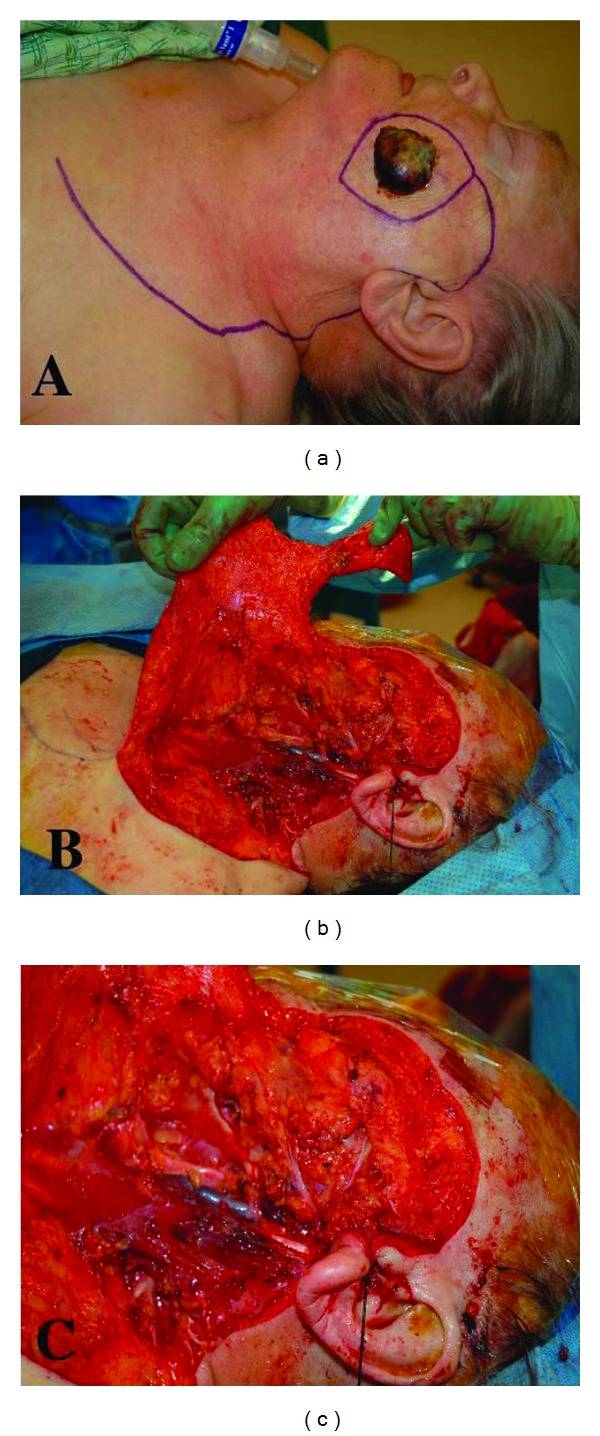
Macroscopic appearance of Merkel cell carcinoma. (a) Surgical photo showing red, violaceous, and firm nodule with a smooth, elevated surface. Markings depict large cervicofacial rotation flap to reconstruct the expected defect. (b) Surgical photo depicting superficial parotidectomy and level I–IV lymph node dissection. (c) Close-up picture of b.

The primary lesion of MCC is distinguished by its absence of distinctive clinical characteristics [4]. In general, MCC occurs more commonly in sun-exposed skin and in elderly individuals. The primary lesion presents as a rapidly growing, asymptomatic, reddish-blue dermal papule or nodule that develops over the course of weeks to months (Figure 1) [4]. The mnemonic AEIOU has been used to describe its clinical appearance and demographic characteristics: asymptomatic, expanding rapidly, immune suppression, older than 50 years, and ultraviolet-exposed/fair skin [5]. Rates of lymph node metastasis can be very high which affect the treatment decisions regarding the neck.

**Figure 2:**
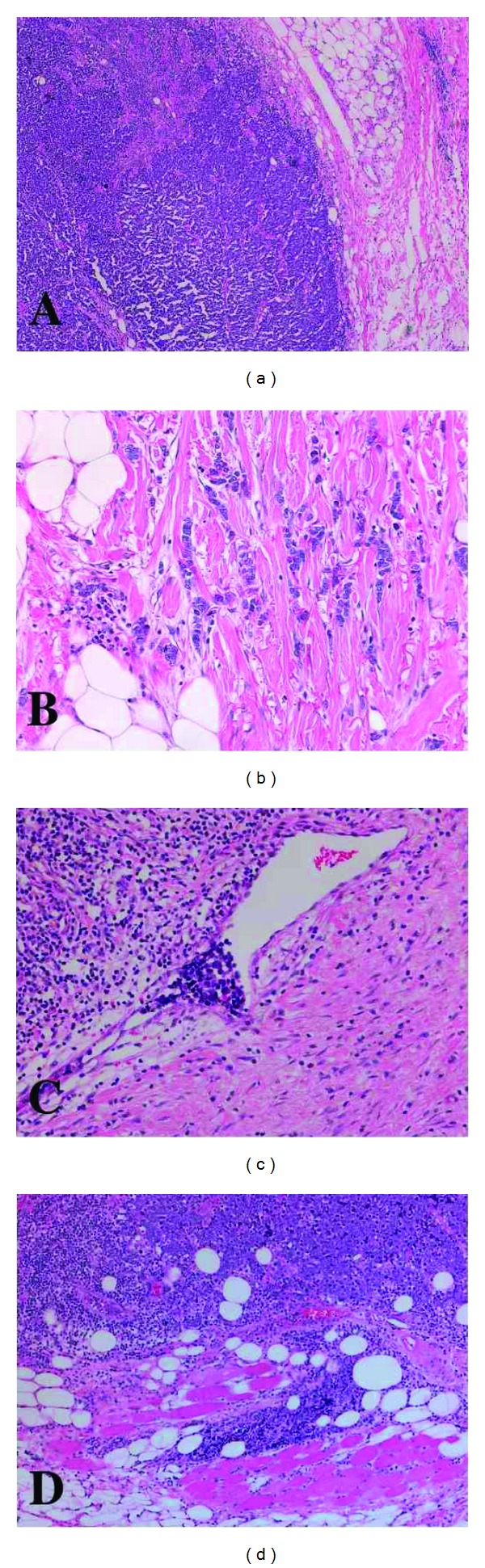
Microscopic appearance of Merkel cell carcinoma. Haematoxylin and eosin staining of a MCC section. (a) Nodular growth pattern. (b) Infiltrative growth pattern. (c) Lymphovascular invasion. (d) Skeletal muscle invasion.

Immunohistochemistry is one of the primary modalities used in the routine diagnostic workup of MCC to help distinguish it from other tumours in the differential diagnosis. CK20 is an intermediate filament protein that has been proposed as the most robust cytokeratin marker for distinguishing MCC from small-cell lung carcinoma and other cutaneous carcinomas [6]. Another biomarker used to differentiate these two carcinomas is thyroid transcription factor-1 (TTF-1). Often, these two biomarkers are used in conjunction because of the rare case of a CK20-negative MCC. There have been no cases of TTF-1 expression in a total of 129 MCC cases studied in the literature [7]. CM2B4 is an antibody that recognizes the Large T (LT) antigen of the Merkel cell polyomavirus (MCPV) and has shown positive reactivity in approximately 70% of MCC [8]. Despite the prominence of immunohistochemistry in the diagnostic workup of MCC, the College of American Pathologists released their 2010 recommendations in the pathological reporting of MCC of the skin. These include type of procedure, tumour site/size, margins, lymphovascular invasion, invasion of deeper soft tissues, and lymph node status (Figure 2).

## 2. Treatment

A plethora of options exist in the treatment of MCC, yet, the optimal option for this aggressive disease has yet to be found. Currently, a multimodality approach is advocated and includes in general a wide and deep local excision with regional lymph node dissection and adjuvant radiotherapy. Radiotherapy as a primary modality has been advocated in cases of inoperable disease. Sentinel lymph node biopsy can help to identify the presence of occult metastatic disease which can have prognostic implications.

## 3. Wide Local Excision

The importance of wide local excision of the primary tumour was shown by Goepfert et al. who found that inadequate surgical excision was a leading cause of local recurrence following radiotherapy [9]. Furthermore, Kokoska et al. found that early, aggressive treatment including surgical excision with margins >2.5 cm resulted in better locoregional recurrence and cumulative survival at 2 years than those patients with simple excision [10]. It was later shown by Allen et al. from Memorial Sloan-Kettering that margins >1 cm were not associated with decreased local recurrence rates [11]. Current recommendations are based on the clinical size of the primary tumour: excision with 1 cm margins for tumours <2 cm, and excision with 2 cm margins for tumours >2 cm [12].

## 4. Sentinel Lymph Node Biopsy

Generally, the progression or “cascade of metastasis” of MCC involves the local disease site, which then travels to regional lymph nodes with ultimate spread to a distant site. In the head and neck, the lymphatic system is very extensive and variable. The sentinel lymph node (SLN) is defined as the first lymph node in a regional lymphatic basin to receive lymph flow from a primary tumour site. The sentinel node is the first lymph node that tumour cells encounter as they spread through lymphatic channels. It is thought that the histologic status of this node predicts the status of the entire regional drainage basin that is at risk for metastases [13]. Therefore, if a sentinel lymph node does not contain metastatic disease, it is unlikely that other nodes in the regional lymph node basin will as well—a finding verified in patients with melanoma [14].

The concept of the sentinel node was first introduced by Cabanas for penile carcinoma in 1977 but has been used more recently in treating patients with melanoma and breast cancer [15]. Unlike in melanoma where histologic characteristics like ulceration and Breslow's thickness can help select patients for sentinel lymph node biopsy, no such characteristics are associated with prognosis that can direct patient management in MCC. Rapid transit time, close proximity of the primary site to the sentinel lymph nodes, spilling of the tracer, and the presence of multiple, contralateral or bilateral sentinel lymph nodes all pose potential difficulties with sentinel lymph node biopsy in the head and neck [16].

As the most consistent predictor of survival in MCC, the status of the regional lymph nodes has garnered much attention. It is controversial whether regional nodal disease is a governor of outcome, but it is certainly a predictor [4]. The biology of MCC is such that regional lymph node metastasis occurs frequently and early in the course of the disease. Nearly one-third of clinically node-negative patients harbour microscopic metastatic disease. Regional node involvement has been reported in up to two-thirds of patients and can be apparent at initial presentation in one-third [17]. It can take up to eight months for nodal metastases to become clinically apparent [17]. Proper identification and staging of the nodal basin could direct treatment algorithms for patients with MCC. These algorithms can include elective neck dissection or adjuvant treatment with radiation or chemotherapy. 

Importantly, prophylactic dissection of the regional lymph node basin is associated with a less than 20% rate of regional failure compared to therapeutic lymph node dissection which is associated with a 60% recurrence rate [18]. This trend mirrors that of melanoma, where only 2% of cases with negative sentinel nodes develop locoregional failure [19]. Ultimately, the staging information provided by sentinel lymph node biopsy can be a primary determinate of ultimate outcome. Five year survival rates for patients with nodal disease are less than 50% compared with 80% in the absence of regional metastasis [20].

A benefit of sentinel lymph node biopsy (SLNB) is that it permits resection of a possible metastasis within the regional lymphatic basin when the tumour burden is likely to be very small [17]. Moreover, this technique can correctly identify the proper nodal basin most likely to harbor micrometastasis rather than relying on traditional anatomic drainage patterns. Up to 20% of melanoma patients undergo nonclassic lymph node dissections based on aberrant lymphoscintigraphy patterns [21].

Sentinel lymph node biopsy is a minimally invasive option in patients presenting with MCC, to avoid the morbidity of elective neck dissection in the 80% of patients who are sentinel node biopsy negative. Mapping should happen at the time of wide local excision, obviating the risk of interruption of cutaneous lymphatics that could result in inaccurate localization of the sentinel node.

In some institutions, when the MCC can be completely excised with negative margins and the sentinel lymph node is negative, adjuvant therapy can be avoided [22]. As in melanoma and breast cancer, sentinel lymph node biopsy has been used to stage the nodal basin in MCC [23, 24]. The rationale for using sentinel lymph node biopsy is based on the similarities between the biology of MCC and malignant melanoma.

One of the first studies using sentinel lymph node biopsy in MCC was published in 1997. Messina et al. studied 12 patients with MCC who underwent removal of a total of 22 sentinel lymph nodes [17]. The two patients with metastatic nodes underwent completion lymph node dissection, while the remaining node-negative patients received no further surgery. The patients with node-negative disease remained free of MCC for a median followup of 10.5 months [17]. Hill et al. performed sentinel lymph node biopsies on 18 patients who underwent removal of 35 nodes [13]. Two patients had metastatic disease in the sentinel lymph nodes and with complete dissection of the nodal basin; no additional lymph nodes were positive, suggesting that the sentinel node had been properly identified [13]. A few years after these studies, Rodrigues et al. published a report on six MCC patients with clinically negative nodes who underwent successful sentinel lymph node biopsy. Three patients had a positive biopsy; all three had systemic chemotherapy and two had adjuvant radiation to the regional lymphatics [23]. Two of the node-negative patients did not have additional treatment and were alive without evidence of disease at 15 month followup [23].

The diagnostic accuracy and usefulness of sentinel lymph node biopsy in MCC have been studied in significant detail. Gupta et al. analyzed 122 patients with clinical N0 staging and found 32% harboured occult metastatic disease, compared to a 5% incidence rate in similarly staged melanoma [25]. As expected, patients with positive SLNB were 3 times more likely to develop recurrent disease than with N0 patients. This study showed that SLNB changed the stage grouping of one-third of MCC patients and in effect altering their treatment course. Many other studies have shown that SLNB can be performed reliably and safely both in the head and neck region [26] and in the extremities [27] to identify occult regional disease.

The importance in addressing the nodal status in N0 disease is highlighted in a recent Australian study that the commonest site of first relapse was in the regional nodal basin. More importantly, 68% of patients with nodal recurrence had stage I disease with untreated nodal basins [28]. This study showed a negative correlation between overall survival and the number of involved lymph nodes [28]. The authors suggest that SLNB could help select those early staged MCCs that could benefit from elective nodal treatment.

A prospective study of sentinel lymph node biopsy in MCC looking at 23 patients showed that accurate staging information can be gleaned by this technique and nodal status does have a differential effect on survival, although this did not reach significance in the study [29]. Tumour foci were found in 11 patients, 50% of which had further positive nodes on completion elective lymph node dissection. Of those patients with a negative sentinel lymph node, 33% relapsed [29]. The authors suggest that negative lymph node biopsy is not necessarily associated with a favourable prognosis and should be used in a diagnostic manner rather than for therapeutic intent. They also observed the histopathological features of the positive lymph nodes and noted that those nodes with tumour foci >2 mm in the sentinel node were more likely to have additional lymph nodes positive [29]. Thus, this technique could identify patients in further need of a complete neck dissection or radiation therapy. Despite treatment, however, the more extensive nodal disease did not seem to have any impact on the ultimate clinical course.

A study from Memorial Sloan-Kettering in New York looking at recurrence and survival in MCC patients undergoing SLNB showed that the only predictors of SLNB positivity were primary tumour size (25% <2 cm versus 45% >2 cm) and the presence of lymphovascular invasion (55% positive versus 4% negative) [30]. Interestingly, they found no difference in recurrence or death from MCC between SLNB-positive and -negative patients. Moreover, only lymphovascular invasion was associated with both recurrence and survival [30].

In a similar study, Schwartz et al. showed a statistically significant correlation between clinical size of the lesion, greatest histologic dimension, tumour thickness, mitotic rate, and growth pattern with SLNB positivity [31]. On multivariate analysis, no models were able to predict a lower than 15% likelihood of SLNB positivity. The authors posit that all patients presenting with MCC without evidence of regional lymph node disease should be considered for SLNB [31].

It is possible that the SLN might not be found due to direct extension from the primary MCC causing emboli and mechanically plugging lymphatic channels. Case reports showing infiltration of both lymph nodes and lymphatic vessels reveal unsuccessful SLNB approaches [32]. In a recent study from the University of Miami, Shnayder et al. reviewed their treatment of MCC. Fifteen patients with MCC were studied, 10 of which have wide local excision and sentinel lymph node biopsy [16]. They were successful in finding the sentinel lymph node in every patient. Those patients who were sentinel node positive received adjuvant radiation. Some of the negative sentinel node patients received radiation as well because of the invasiveness of the primary site.

Mehrany et al. performed a meta-analysis of the prognostic significance of sentinel lymph node status in MCC [33]. They reported data on 60 patients, 40 of whom had a biopsy-negative sentinel lymph node. Thirty-five of these patients had no further treatment and the remaining had completed neck dissection and adjuvant radiation or adjuvant chemoradiation [33]. One patient in this group died of metastatic disease, while the remaining patients had no recurrence at a mean of 7.3 months. The other 20 patients had biopsy-positive sentinel lymph nodes, with 15 having additional treatment. Three of the remaining five patients developed regional nodal recurrence. The risk of recurrence or metastasis was 19-fold greater in the biopsy-positive patients [33]. Only one patient with a negative sentinel lymph node experienced disease recurrence. The study authors concluded that sentinel lymph node positivity was a strong predictor of high short-term risk of recurrence and that completion of neck dissection was beneficial in alleviating this risk [33].

Warner et al. looked at their group of 11 patients who had sentinel lymph node biopsy of whom 3 were positive [34]. Two of these patients developed recurrence despite surgery and radiation. Of the eight patients who were sentinel lymph node negative, five developed recurrence of the disease [34]. This high percentage, 67%, is much higher than the average 30% seen in other studies.

They also found no correlation between depth of invasion and sentinel lymph node biopsy positivity. 

Immunohistochemical analysis of sentinel lymph nodes from patients with breast carcinoma or melanoma increases the sensitivity for detection of metastases in up to 40%. Up to 40% of patients with occult MCC nodal metastasis will be missed if evaluation is limited to standard hematoxylin-eosin (H&E) staining [35, 36]. In MCC, this question was addressed by Allen et al. who studied 26 patients and found that 2 out of 5 patients with lymph node metastases were identified only after confirmation with immunohistochemical staining [37]. The addition of immunohistochemistry improves the ability to identify those patients with regional micrometastatic disease, known as “minute metastases” otherwise undetectable using traditional H&E staining. 

Schmalbach et al. studied 10 patients with MCC who had sentinel lymph node biopsy and found that the two patients with positive lymph nodes appeared negative on hematoxylin-eosin staining. MCC was identified using CK20 immunostaining [38]. It should be noted that the clinical significance of submicroscopic lymph node metastases identified only by immunohistochemistry remains unclear.

Sentinel lymph node biopsy involves very little morbidity and can be used to stage the disease. In some patients, this technique helps avoid the risks of complete lymph node dissection and in others can direct further management decisions. The sentinel lymph node biopsy has the advantage of providing the pathologist with only a few samples, allowing a thorough slice-by-slice histopathological analysis. This extensive pathological evaluation would be impossible in neck dissection samples, where up to 30 lymph nodes can be included.

The high rate of regional metastases and associated poor prognosis provide an impetus to treat regional lymph node basins, like the neck, in a prophylactic manner. Although there may be a benefit in regional control and disease-free survival using elective neck dissection compared to therapeutic neck dissection, there are no reports in the literature showing any survival advantage.

Identification of any positive sentinel lymph nodes makes the initial procedure a staging one and then should be followed by a formal lymph node dissection or by adjuvant radiotherapy, especially in head and neck MCC. Patients, however, are often unwilling to undergo a second intervention [16, 29]. There remains the option of upfront elective lymph node dissection using the gamma probe as a guide. This approach would provide therapeutic treatment of the regional lymph node basin and prevent missed nodes as a result of aberrant drainage patterns. Within the head and neck, lesions in the midline may drain to either side of the neck or parotid gland.

Another option for those patients unwilling to undergo formal lymph node dissections upfront would be performing sentinel lymph node biopsy and relying on immediate frozen section results to dictate further management. A positive result would lead to immediate completion of lymphadenectomy. Patients would need to be informed about this and counseled about the probability of further treatment if there was metastatic disease identified on final pathology or immunohistochemistry.

## 5. Conclusion

Merkel cell carcinoma is a rare and aggressive cutaneous neoplasm. Advances in immunostaining are aiding in the diagnosis of this disease. With the discovery of the polyoma virus, a great deal of interest should be placed in reevaluating the role of radiotherapy in treating those virus-positive patients. Furthermore, the indications for sentinel lymph node biopsy are still being elucidated and vary between institutions. Clearly, a multidisciplinary approach to this disease is required, and the next decade should provide more insights into the best treatment of this rare disease.
